# Aldose reductase, fructose and fat production in the liver

**DOI:** 10.1042/BCJ20240748

**Published:** 2025-02-19

**Authors:** Peter Delannoy, Dean R. Tolan, Miguel A. Lanaspa, Iñigo San Millán, So Young Bae, Richard J. Johnson

**Affiliations:** 1 Orgins of Human Metabolic Disease, Phoneix, AZ,85016, U.S.A; 2 Department of Biology, Boston University, Boston, MA, U.S.A; 3 Division of Endocrinology, Metabolism and Diabetes, University of Colorado Denver, Aurora, CO, U.S.A; 4 Department of Medicine, Division of Endocrinology, Metabolism and Diabetes, University of Colorado Anschutz Medical Campus, Aurora, CO 80045, U.S.A; 5 Molecular Biology, Cell Biology, and Biochemistry Program, Boston University, Boston, U.S.A; 6 Department of Medicine, University of Colorado Anschutz Medical Campus, Aurora, CO 80045, U.S.A

**Keywords:** aldose reductase, fructose, glucokinase, polyol pathway, sorbitol, warburg

## Abstract

There is an increasing interest in the role of fructose as a major driver of non-alcoholic fatty liver disease (NAFLD), and it is linked closely with the intake of sugar. However, there has also been the recognition that fructose can be produced directly from intracellular glucose via the evolutionarily conserved polyol pathway whose access is governed by aldose reductase (AR). The purpose of this article is to review the biochemistry of AR and the role of the polyol pathway in opening fructose metabolism. This article provides a new perspective about AR and the other key enzymes surrounding the decision to divert glucose into the polyol pathway which suggests that the production of endogenous fructose may be of much greater significance than historically viewed. There are important aspects of the regulation of the polyol pathway and its committal step catalyzed by AR, which supports the notion that fructose-uric acid pathway is activated by elevated glucose with the downstream consequence of NAFLD and perhaps other chronic metabolic diseases.

## Introduction

The polyol pathway is the only known way that glucose can be converted to fructose in humans, and it appears to be ubiquitous in all tissues where it has been rigorously looked for, along with the tissue-specific activation of fructose-uric acid metabolism [1,2]. The role of glucose in the formation of endogenous fructose via the polyol pathway has been demonstrated in animals and humans and is found throughout human tissues including the liver, kidney, and brain [3–5]. The polyol pathway, its key enzyme, aldose reductase (AR; AKR1B1), and its over expression has been implicated in many disease states, including diabetes and its complications, diet-induced obesity and metabolic disease, neurological disorders including dementia, behavioral disorders and multiple sclerosis, certain cancers, and other conditions such as tissue ischemia [3,6]. The participation of the polyol pathway and AR in so many diverse conditions has led to questions about its regulation and function. Previous reviews have even suggested that the ‘natural’ substrate of AR and its physiological role remains unknown (other than its constitutive role in urinary concentration in the renal medulla) [7–9].

One potential answer to this paradox may lie in the specific way in which the enzyme AR is regulated under different physiological demands and conditions [6–13]. For example, AR is known to reduce aldose sugars like glucose and aldehydic lipid peroxidation products to the corresponding alcohols. Examples include 4-hydroxynonenal, glyceraldehyde, retinoids, and methyl glyoxal in which the Km values range between 8 and 50 uM [6,8]. In contrast, the Km values for glucose have been measured from~150 mM to as low as 0.66 uM [14–16]. It could be that the normal Km in the unactivated state of AR is quite high but with an allosteric shift to the activated state there is a marked change in affinity leading to activation at glucose levels that are commonly observed in both diabetes and normal individuals postprandial to a high carbohydrate meal. In this review, we discuss the nuances of this regulation and suggest how AR may open the polyol pathway and drive endogenous fructose production and fat synthesis in the liver.

**Figure 1:**
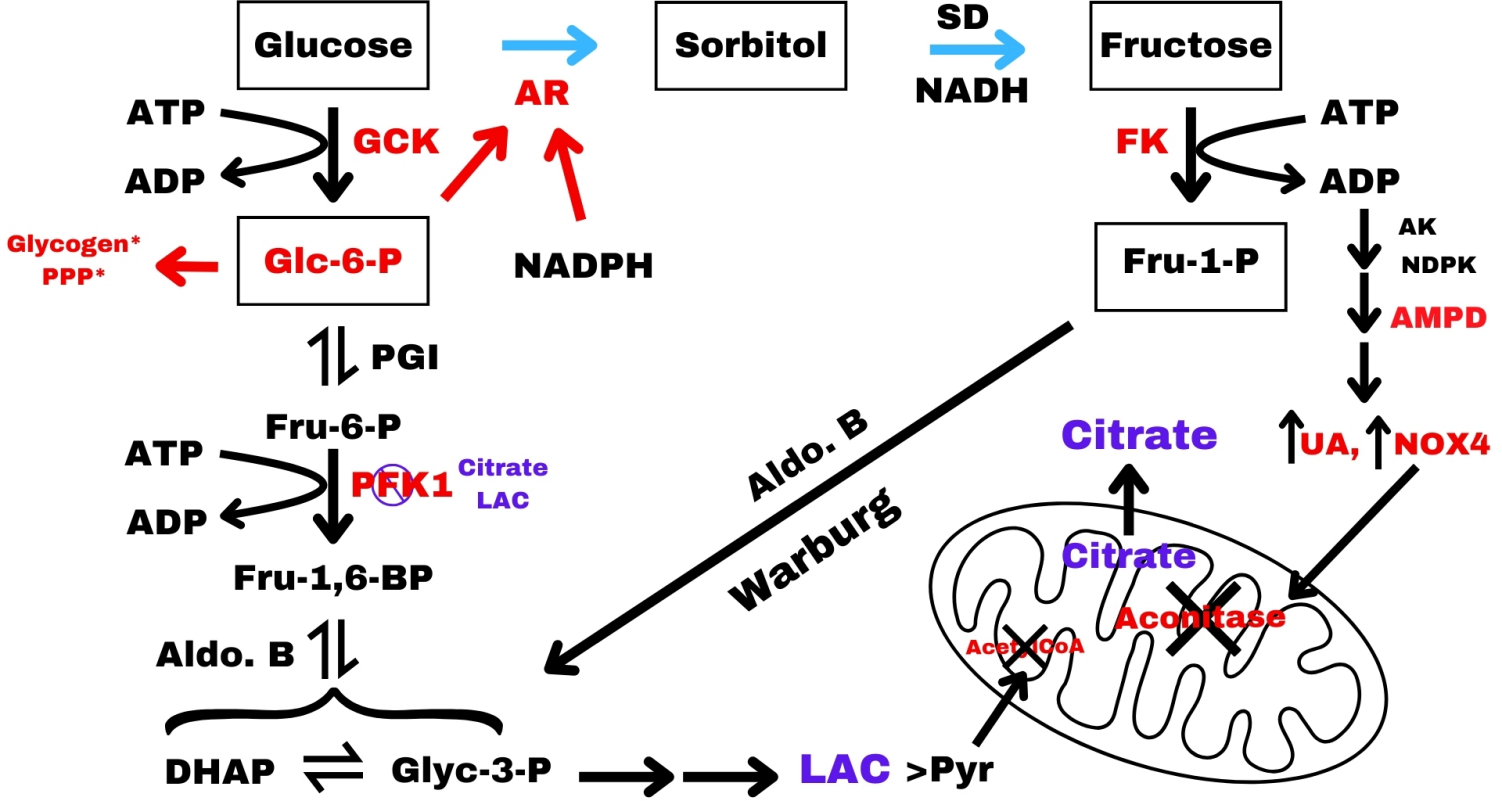
Polyol-fructose-uric acid pathway in the liver. A condensed version of glycolysis, polyol pathway (blue arrows), and the Warburg effect where the fructose-uric acid pathway reconnects with glycolysis below the PFK1 step. The polyol pathway is shown by blue arrows. Regular glycolysis proceeds below Glc6P via the black arrows to pyruvate. The inhibition of mitochondrial acetyl-CoA formation during fructose metabolism is noted by the black X. The inhibition of aconitase by NOX4, as well as the accumulation of cytoplasmic lactate and citrate and their inhibitory effect on PFK1, is diagramed. The allosteric effect of NADPH and Glc6P on AR is denoted by the red arrows. The upregulation of NOX4 by UA is indicated by the black arrow. NOX4 is then translocated to the mitochondria where it inhibits aconitase with the result that citrate is shuttled into the cytoplasm. Glycogen* synthesis and the pentose phosphate pathway (PPP) are only indicated by a red arrow in the upper left of the figure. GCK, glucokinase; AR, aldose reductase; SD, sorbitol dehydrogenase; FK, fructokinase; AK, adenylate kinase; NDPK, nucleotide diphosphate kinase; AMPD, AMP deaminase 2; UA, uric acid; LAC, lactate; Pyr, pyruvate; DHAP, dihydroxyacetone phosphate; Glyc-3-phosphate, glyceraldehyde-3-phosphate; Aldo. B, aldolase B; HK, Hexokinase; F-1,6-BP, fructose-1,6-bisphosphate; PFK1, phosphofructokinase 1; PGI, phosphoglucose isomerase; PPP, pentose phosphate pathway; ChREBP, carbohydrate response element-binding protein.

In the liver, the activation of the two-step polyol pathway is governed by the enzyme AR (Figure 1; [3]). Previous *in vitro* biochemical analyses in preparations of erythrocytes, aorta, brain, muscle, and bovine lens have reported AR Km values for glucose ( < 10 mM) which is in a similar range to the Km values reported for glucokinase (GCK; ~ 7 mM) [15,17–20]. This means that AR and GCK might be activated under similar glucose concentrations in the liver. Early studies have shown that *in vitro* purified AR exists in two reversible forms: the inactivated and activated state where the shift to the activated state requires the binding of glucose, glucose-6-phosphate (Glc6P), and NADPH [15,17,18,21]. Moreover, the unactivated and activated forms of AR show markedly different characteristics where the unactivated enzyme displays biphasic kinetics (Km values of 9 mM and 0.9 mM) and the activated form of AR displays monophasic kinetics (Km value of 0.68 mM) [15,17]. In addition, the activated enzyme is less responsive to AR inhibitors which is consistent with a change in conformation to the activated state and may explain the change in its Km value for glucose [15,17].

**Figure 2:**
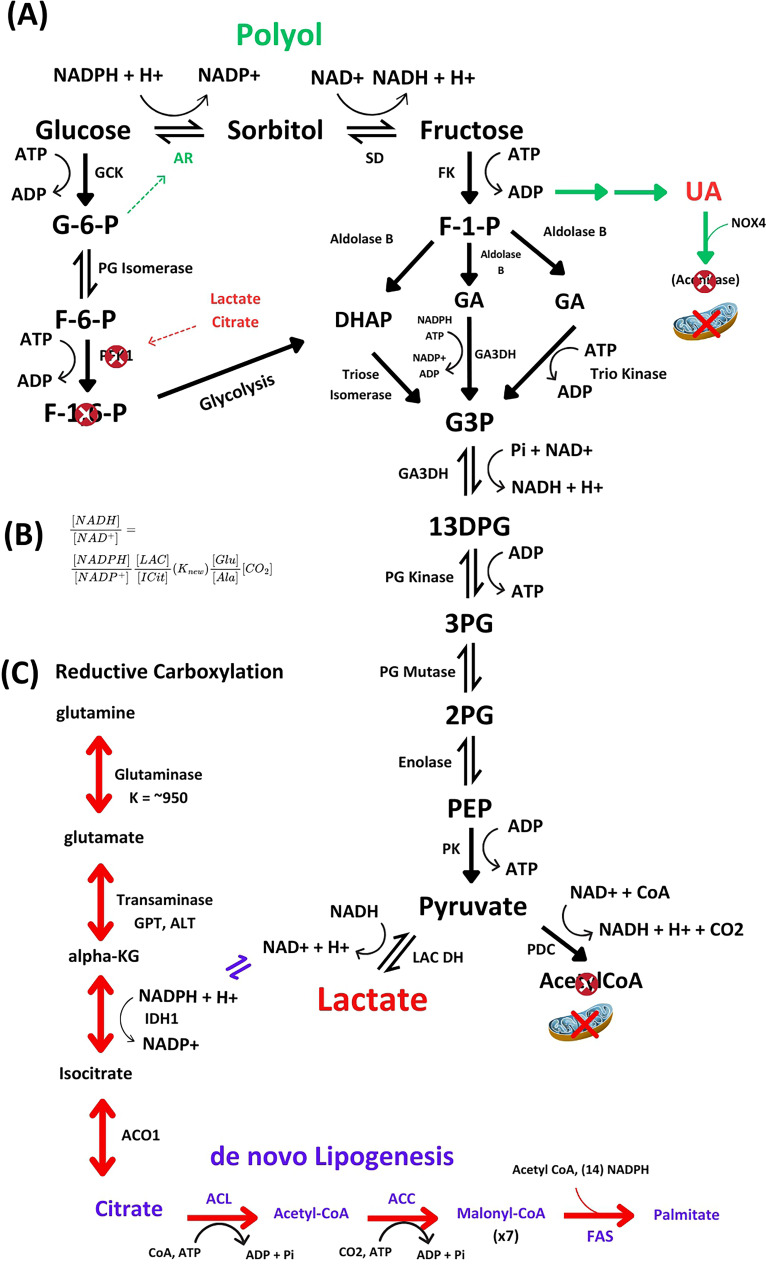
The complete polyol-fructose-uric acid pathway to lactate and the connection to reductive carboxylation in the context of aconitase inhibition. **(A)** Polyol-fructose-uric acid pathway favoring lactate and inhibition of the PDC. Normal pathway glycolysis is shown by the black arrow from F-1,6-P to DHAP. **(B)** The *in vitro* determined equilibrium expression linking LDH to IDH1 first proposed and experimentally determined by Veech, R.L. et al. (1969). **(C)** The possible formation of cytosolic citrate by glutamine and reductive carboxylation. The pentose phosphate and glycogen synthesis connections are shown in Figure 3.

**Figure 3:**
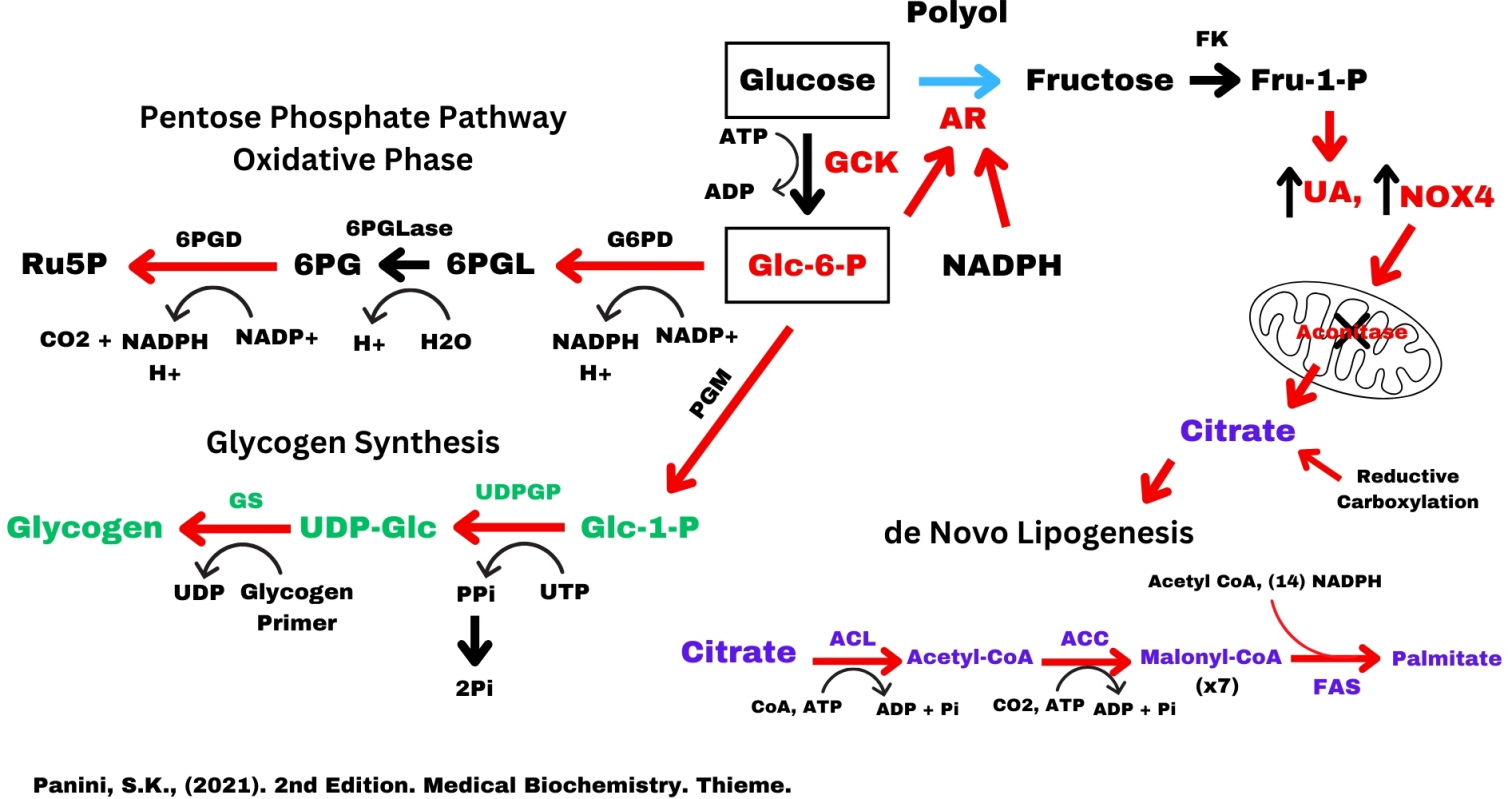
This figure shows the abbreviated pentose phosphate pathway (PPP), glycogen synthesis, and *de novo* lipogenesis (DNL) and the possible contribution to citrate from reductive carboxylation. All three of these pathways are activated during polyol-fructose-uric acid biochemistry and the elevated concentration of Glc6P. FK, fructokinase; G6PD, glucose-6-phosphate dehydrogenase; 6PGLase, 6-phosphogluconolactone hydrolase; 6PGD, 6-phosphogluconate dehydrogenase; PGM, phosphoglucomutase; PPP, pentose phosphate pathway; UDPGP, UDP-glucose pyrophosphorylase; GS, glycogen synthase; ACL, ATP citrate lyase; ACC, acetyl-CoA carboxylase; DNL, *de novo* lipogenesis; FAS, fatty acid synthase.

GCK produces elevated concentrations of Glc6P and has a preference for the alpha anomer of glucose [16,22]. This suggests that GCK and AR could work together to open the polyol pathway for two important reasons. First, GCK produces elevated Glc6P which is known to operate on AR with NADPH and glucose to shift AR to the activated state. Second, Inagaki et al. have shown that AR has a preference for the aldehyde form glucose (Km 0.66 uM) and that the rate of reduction to the alcohol is faster from the cyclic alpha anomer of glucose than from the beta anomer of glucose by AR [16,22]. Therefore, the fact that AR seems to be more efficient with the cyclic alpha anomer of glucose could play a role in the reduction in glucose to sorbitol in the first step of the polyol pathway. However, it is important to mention that the Inagaki et al. study [16] did not differentiate between the unactivated and the activated forms of AR and more work is needed to understand the relationship between this study and how it relates to the activated state of AR and the activation of the polyol pathway (Figures 1 and 2).

## The polyol pathway is a bridge between two metabolic options in the liver

The polyol-fructose-uric acid pathway may form an integrated system that modulates the oxidation of glucose via glycolysis and the pentose phosphate pathway (PPP) for energy and metabolites versus the synthesis of fat and glycogen governed by the activation of AR in the liver. The bridge between the two states is the polyol pathway that connects glycolysis and fructose-uric acid metabolism as shown in Figure 1 [3, 23 and 24].

When the polyol-fructose-uric acid metabolism is opened, there is an acute rise in intracellular uric acid with a shift in flow of metabolites around the PFK1 step of glycolysis through fructose-1-phosphate to DHAP and glyceraldehyde with a Warburg effect that results in the increase in the lactate to pyruvate ratio (Figure 2; [3,23]). Concomitantly, the rise in intracellular uric acid induces the translocation of NADPH oxidase (NOX4) to the mitochondria where the oxidative stress inhibits aconitase in the TCA, resulting in the elevation of cytoplasmic citrate [24,25]. This results in a shift in metabolite flow through polyol-fructose-uric acid into *de novo* lipogenesis (DNL) (Figures 2 and 3) rather than oxidation via the TCA cycle or the PPP. At the core of this decision to choose fat synthesis or oxidation with ATP production may be a synergistic relationship between the two enzymes, AR and GCK, whose activation is ultimately dependent on the concentration of glucose. This leads to the question of how AR is regulated.

**Figure 4:**
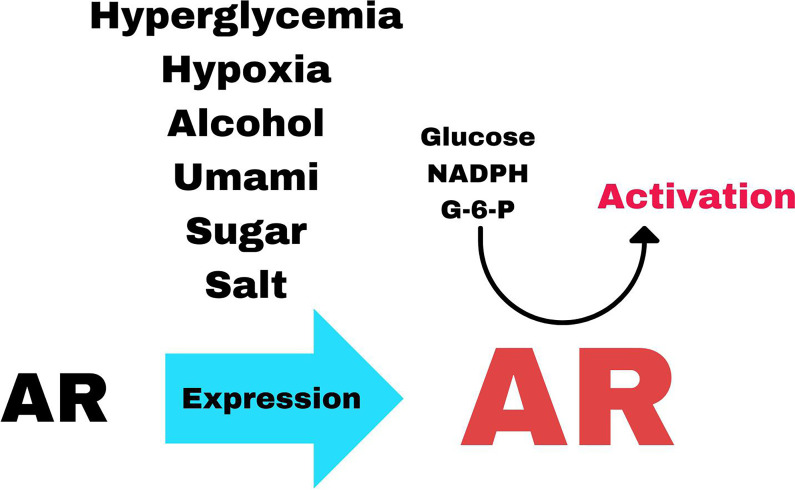
The substances that increase the expression of aldose reductase (AR) including the specific enzymatic activators of AR.

Specifically, hyperglycemia, fructose, added sugar (glucose and fructose), alcohol, umami (processed food and meat), and stress (hypoxia, dehydration, salt, and hyperosmolality) induce the expression of AR (Figure 4; [3,23,26]). Note that the elevation of AR due to increased expression means that under the pressure of the above metabolites, there is more AR available to be allosterically activated and subsequently to open the polyol pathway. It has been previously shown that the opening of the polyol pathway is dependent on the allosteric activation of AR by Glc6P and NADPH (Figure 4; [15,18]).

## Under low glucose conditions GCK and AR are inactive and the polyol pathway is closed

**Figure 5:**
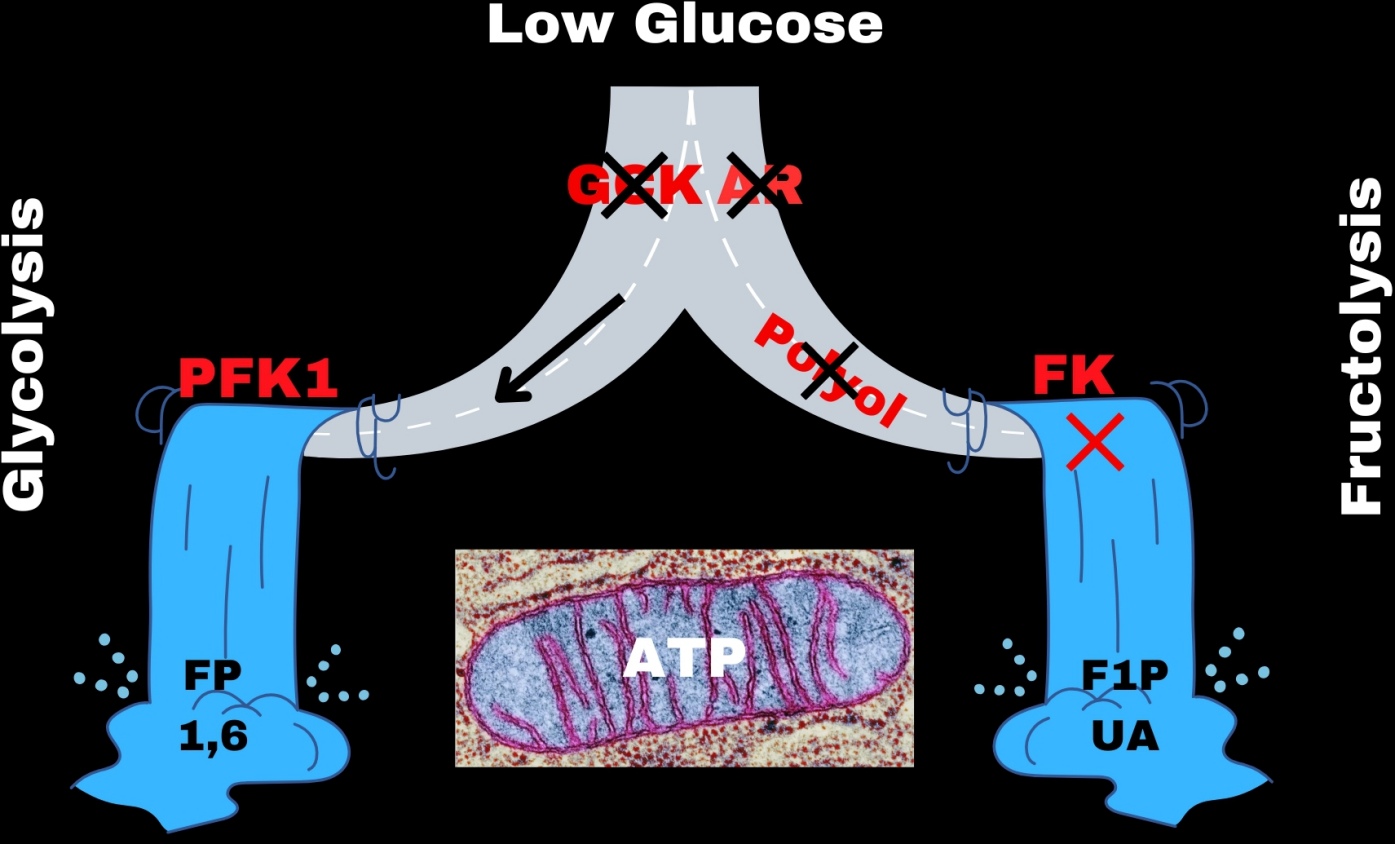
The mechanics of the switch between glycolysis and polyol-fructose-uric acid. The gateway enzymes, glucokinase (GCK), and aldose reductase (AR) are shown in red. The left fork gives glycolysis and the right fork gives fructose-uric acid metabolism depending on the concentration of glucose and the activation or inactivation of GCK and AR.

To better understand this relationship, we first consider the intracellular glucose conditions where AR and GCK are in an inactivated state. The key enzyme(s) responsible for the first step of glycolysis under low glucose conditions (under 5 mM glucose) is hexokinase ([27–30]; I, II, III, and IV (GCK) depending on the tissue). Under low glucose conditions, GCK and AR are inactive (Figure 5). The hexokinase enzymes (I, II, III) have a low Km value (~0.1 mM), a high affinity for glucose, catalyzes the conversion of glucose to Glc6P, and is mostly saturated at all physiological concentrations of glucose and is strongly inhibited by Glc6P [27–30]. Furthermore, the reaction has a strong thermodynamic driver where the change in free energy is approximately −17 (kj/mole) resulting in a K_eq_ value that is over 800 [31,32]. The concentration of Glc6P rises in accordance to the glucose concentration.

Collectively, the hexokinases provide tissues with glucose at a rate that depends on the cellular demand for ATP and maintenance of the phosphorylation potential [20,33,34]. The rapid conversion of glucose to Glc6P serves to maintain a steep concentration gradient favoring the entry of glucose into the liver from the portal vein via GLUT2 and to maintain a continuous flux of substrate into glycolysis and subsequent flow of substrate into the mitochondria for pyruvate oxidation. Under these conditions, GCK and AR remain inactivated and only about 3% of the total glucose is converted into sorbitol and then into endogenous fructose via the polyol pathway [2,27,28].

## High glucose activates AR and GCK, and polyol-fructose-uric acid is opened

**Figure 6:**
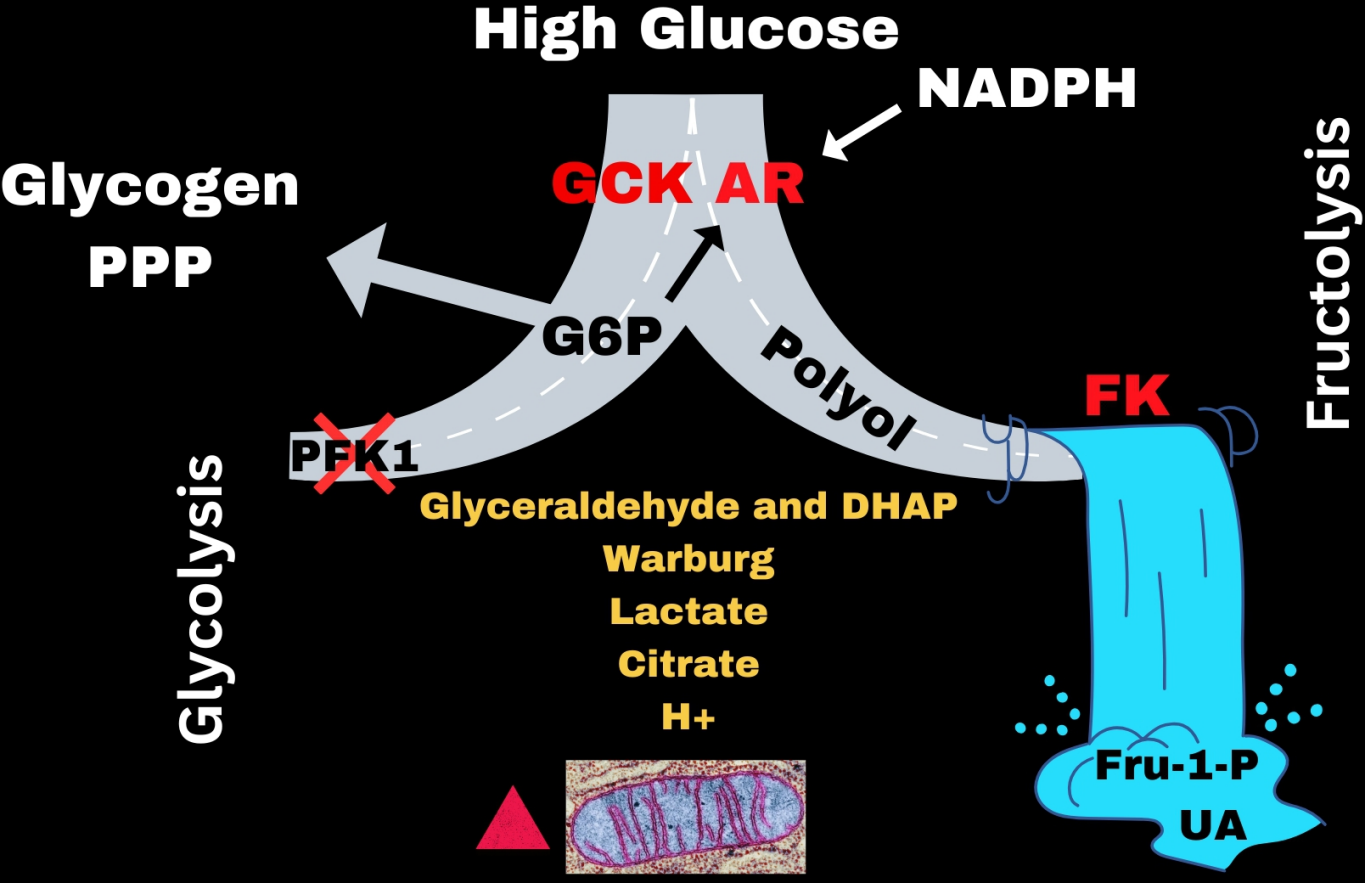
Activation of the gateway, GCK and AR, and the subsequent opening of fructose-uric acid metabolism.

However, as blood glucose rises above ~ 5 mM (90 mg/dL), like it would after a meal rich in carbohydrate, GCK and AR may be quickly activated and work synergistically to open the polyol pathway enabling the cascade to fructose-uric acid metabolism in the liver ((Figure 6) [2,15,19,20]). Under these glucose conditions, the glucose sensor, GCK is released from glucokinase regulatory protein (GKRP) in the nucleus and translocates to the cytoplasm where it mediates the conversion of glucose to Glc6P [19,20]. GCK is prevented from re-associating with the glucokinase regulatory protein (GKRP) by micromolar concentrations of fructose, sorbitol, and Fru1P [19,20], which means that once the polyol pathway is opened, it maintains active GCK and the subsequent supply of Glc6P. The overall activation of GCK is heavily influenced by the early products of the polyol-fructose-uric acid pathway and fructokinase. Literally, there is a 1000-fold difference in magnitude between the glucose effect and the fructose effect on GCK activation in the liver.

Under these conditions, AR is activated and up to 30% of the intracellular glucose is converted into endogenous fructose via the two-step polyol pathway where glucose is first reduced to sorbitol by AR and then oxidized to fructose by sorbitol dehydrogenase [2,15,35]. The induced expression of AR by the substances illustrated in Figure 4 could have the additive effect of enhancing this reaction by increasing the concentration of enzyme that can be shifted to the activated state and stimulate the polyol pathway.

Moreover, once AR is activated, the change in potential energy of the combined coenzyme couples, [NADPH]/[NADP+] and [NAD+]/[NADH], drive the flow of glucose to endogenous fructose [33,36]. Supporting this argument is the understanding that the conversion of glucose to fructose through polyol involves two reactions with equilibrium constants near unity and that the coupling with [NADPH]/[NADP+] and [NAD+]/NADH] coenzyme couples provide the driving force necessary to yield the quantitative conversion of glucose to fructose by this *in vivo* reaction [36].


$$glucose+NADPH+NAD+\to NADP^{+}+NADH$$

There are several notable aspects about this coupled reaction: (1) The main driving force is provided by the reduction in NAD+ by NADPH. (2) NADPH is classically involved in biosynthetic sequences and NAD+ in degradative processes. Here, both are combined in sequence where NADPH opens the very first reaction that drives the eventual cascade to DNL and glycogen synthesis consistent with its role in cellular biosynthetic pathways, and ensures the polyol pathway, as well as DNL, only operate when NADPH is high. (3) Once AR is activated, the reaction sequence flows from glucose to fructose unabated and suggests that AR serves as an enzymatic gateway. In essence, the flow of polyol through fructose uric acid is controlled by a kinetic gateway enzyme: AR.

Under the conditions that activate AR, the increased Glc6P also serves to open the synthesis of glycogen and the PPP (Figure 3; [37,38]). The elevated Glc6P provided by GCK allosterically activates glycogen synthase, increases the enzyme’s affinity for UDP-glucose, and promotes glycogen synthesis [38]. Additionally, elevated lactate also stimulates the PPP and up-regulates NOX4 in chondrocytes [39]. Activation of the PPP produces the NADPH required for the allosteric activation of AR and drives the downstream nucleotide synthesis and the polymerization of glucose to glycogen.

## The fructose-uric acid cascade: fructokinase, AMPD, uric acid and lactate

Once the polyol pathway begins to produce endogenous fructose in the liver, fructokinase immediately, and without feedback inhibition, phosphorylates fructose to Fru1P (Figures 1 and 2). Early *in vitro* studies in rat liver homogenates and recent *in vivo* studies have shown that this reaction is fast and that the accumulation of Fru1P happens quickly after the hepatocytes are exposed to fructose [40,41]. Additionally, this kinase reaction occurs at a rate that is 10 times the rate of the GCK phosphorylation of glucose [42] and shares a change in free energy that is nearly identical to the change in free energy for the reaction catalyzed by phosphofructokinase 1 (~−14.4 kJ/mole (PFK1) and −14.2 kJ/mole (FK) with equilibrium constants of approximately 330 and 311, respectively [32,43,44].

The fructokinase reaction is both fast and irreversible but is not regulated like PFK1 ([45,46]; PFK1 is inhibited by citrate and lactate; see below). Fructokinase drives a cascade that results in a sudden acute decline in both cellular ATP and GTP, as well as phosphate, resulting in an acute sudden rise in uric acid via the induction of AMP deaminase (AMPD) [25,47–49]. The sudden decline in GTP and phosphate activates AMPD, which initiates the purine degradation pathway and rapidly produces uric acid ([25,48]; See Figure 1a). This ‘energy-depletion’ pathway is key to the effect fructose has in the liver and operates quickly to maintain the cell’s energy charge (ratio of ATP to AMP) as ATP drops, so does AMP [48–50]. The fall in ATP in response to fructose can be measured *in vivo* in human liver by magnetic resonance spectroscopy [51]. In addition, the activation of AMPD down-regulates AMP kinase (AMPK) and drives fatty acid accumulation by deactivating acetyl-CoA carboxylase 2 along with the simultaneous up-regulation of carbohydrate response element-binding protein (ChREBP) [25,52–54].

In complement with this process, the rise in uric acid is strongly associated with an increase in the expression of NOX4 and its translocation to the mitochondria where aconitase, a key enzyme of the Krebs cycle, is inhibited by NOX4-generated ROS resulting in the pooling of citrate which is then shuttled out of the mitochondria into the cytoplasm and initiates DNL [24,25,52,55,56]. Uric acid has been shown to induce the expression of NOX4, increase mitochondrial oxidative stress with an increase in ROS, alter the morphology of the mitochondria, and reduce the expression of aconitase in several human cell lines [24,55,57,58], as well as *in vivo* [24,59]. The down-regulation of AMPK, activation of ChREBP signaling, activation of NOX4, and cytosolic citrate initiate DNL. In addition, fructose degradation by the gut microbiome also generates a sizable amount of acetate that can be converted to acetyl CoA to provide additional substrate for DNL [60].

## DNL and the drive to NAFLD

The transcription of both ChREBP and SREBP-1c are up-regulated by fructose-uric acid pathway and may synergistically activate the transcription of the key DNL enzymes including ATP citrate lyase, acetyl-CoA carboxylase 1, fatty acid synthetase, and steroyl-CoA desaturatase 1 and may induce the low-density lipoprotein (VLDL) export of lipids by regulating microsomal triglyceride transfer protein [24–61]. Additionally, ChREBP is activated by the polyol pathway and fructose exposure, and elevates Glc6P, ribose-5-phosphate, and xylulose-5-phosphate consistent with the stimulation of the PPP pathway (Figure 3) [61–62]. ChREBP has different isoforms based on pre-mRNA splicing, each with different roles, but data show that ChREBP-beta is a sensor for fluctuations in glucose and fructose and is strongly linked to ATP and phosphate homeostasis in the liver and also in other organs [41–63]. ChREBP also regulates the transcription of fibroblast growth factor 21 (FGF21) which is known to regulate carbohydrate intake [61]. In addition, ChREBP may induce inflammation, as it caused the activation of NLRP3 inflammasome in a murine diabetic kidney disease model [64]. Importantly, ChREBP-dependent DNL results in the formation of oil droplets (triglycerides) in the liver that are packaged into VLDL and released into the circulation, where the elevation of diacylglycerols, ceramides, acyl-carnitines, and fatty acids is known to be associated with insulin resistance [3,24,62,65–67]. Fructose drives a significant change in the amount, composition, and proportions of fats produced in the liver that can lead to lipotoxicity and NAFLD [66,67].

## Uric acid signaling drives a change in mitochondrial function

The inhibition of aconitase by oxidative stress induced by uric acid enhances the release of citrate into the cytoplasm where it initiates the synthesis of DNL. The elevation of citrate in the cytoplasm inhibits PFK1, redirecting fructose-6-phosphate to glycogen synthesis, PPP, and the polyol pathway via a mass action shift in equilibrium to Glc6P. Moreover, the acute rise in uric acid also inhibits enoyl-hydratase, a key enzyme required for beta-oxidation of fatty acids in the matrix of the mitochondria [24,52,68]. The stimulation of DNL results in an increase in malonyl-CoA concentration which inhibits carnitine palmitoyltransferase 1 (CPT1) and prevents acyl-CoA’s from entering the mitochondria, where, under low glucose conditions, the acyl-CoAs would have been processed by beta-oxidation [24,52,68]. The combined effect may be to dampen fatty acid beta oxidation and to initiate the synthesis of fats in the cytoplasm. The malonyl-CoA inhibition of acyl-CoA entry into the mitochondria occurs, as we will see below, in synergy with a fructose-derived lactate effect due to the accelerated fructolysis. The resulting Warburg effect results in the production of lactate where the lactate:pyruvate ratio is increased above ~ 7:1 [23,69–71]. The elevation in the lactate-to-pyruvate ratio correlates with a decrease in the NAD+/NADH ratio that occurs with the flow of substrate through fructose-uric acid pathway. This results in the NADH inhibition of the pyruvate dehydrogenase complex (PDC) and subsequent redirection of pyruvate to lactate rather than pyruvate to acetyl-CoA [38,72]. Moreover, activation of fructose-uric acid pathway induces the expression of HIF1 alpha which down-regulates PDC and stimulates reductive metabolism of glutamine and alpha-ketoglutarate to synthesize cytosolic citrate and acetyl CoA to support DNL ([73–75]; (Figure 2)).

## Activation of polyol-fructose uric acid drives a lactate effect

Lactate, the mandatory end product of glycolysis, functions as both an energy substrate and a signaling molecule with endocrine, paracrine, and autocrine properties under various glucose conditions [69,70]. Under accelerated glycolysis, there are three proposed lactate functions that are part of the fructose cascade [69,70]: (1) Lactate binds to and inhibits carnitine palmitoyltransferase I and II (CPT I and II) working in synergy with malonyl-CoA to prevent oxidation of fatty acids in the mitochondria. (2) Lactate is transported out of the liver into the circulation system by a symport, monocarboxylate transporter I (MCT1) in a ratio of 1:1, increasing extracellular hydrogen ion concentration (lactic acidosis) [76]. Furthermore, circulatory lactate binds to GPR81 receptors on adipocytes inhibiting lipolysis [77] synergistically with the insulin effect and to the kidneys where lactate can drive gluconeogenesis. (3) Elevated lactate alters the composition of cardiolipin which is highly integrated into the electron transport chain and also influences other enzymes involved in fatty acid oxidation. Previously published data suggest that cells with altered cardiolipin are deficient in respiration and oxidative phosphorylation and can change mitochondrial function [69,70,78,79]. This cascade, triggered by the activation of AR, drives a systemic change in mitochondrial function along with a shift to inflammation and the alteration of insulin signaling. Finally, exacerbated rate of triose metabolism and mitochondrial dysfunction leads to increased production of lactate [80]. Moreover, it is known that the alteration in nutrient conditions alters the [NAD+]/[NADH] coenzyme couple and that this ratio decides direction and extent of metabolic pathway decisions, the choice between lactate versus acetyl-CoA [33]. Early studies have shown that the lactate:pyruvate ratio depends on the flux of metabolites through glycolysis and can vary from~4:1 to over~20:1 [69,80,81]. This suggests that under chronic conditions of hyperglycemia that the shift to lactate versus acetyl-CoA along with the other effects described herein may result in mitochondrial dysfunction over time and are dependent in part on the [NAD+]/[NADH] coenzyme couple.

## Activation of polyol pathway and fructose-uric acid pathway drives systemic inflammation

Fructose-uric acid metabolism has been shown to induce the up-regulation of JNK1 and IL-1beta promoting systemic inflammation and insulin resistance [24]. The elevation of uric acid induces the expression of HIF-1 alpha and down-regulates urea cycle enzymes which also increases hepatocyte inflammation and oxidative stress [82]. Along with urea-cycle inhibition, there is an inhibition of nitric oxide synthase with the downstream effect of altering nitric oxide signaling and a change in expression from KHK-C to KHK-A [26,82–85].

## The polyol pathway and fructose-uric acid pathway alters insulin signaling in the liver

Activation of these two pathways drives a decrease in the density of the insulin receptor on the surface of the hepatocyte plasma membrane and a decrease in insulin receptor substrate 2 (IRS2) [24]. These two effects may alter liver insulin signaling and extraction since insulin extraction depends on the interaction of insulin with the insulin receptor at the surface of the hepatocyte [86–88].

Up to 80% of the insulin released by the pancreas is extracted by the liver and the loss of the insulin receptor on the plasma membrane may decrease the amount of insulin extracted which then could drive hyperinsulinemia downstream in the peripheral tissues [86–88]. In short, the activation of AR and the polyol-fructose-uric acid pathway drives systemic effects that not only promotes the synthesis of fat and glycogen but also systemic inflammation and insulin resistance. The gateway to the flow is AR.

## Summary

Taken together, the biochemical literature suggests that the relationship between glucose and AR is complex and may be allosterically activated by Glc6P and NADPH under conditions of elevated glucose. GCK is also activated under similar glucose conditions and likely provides the Glc6P in appropriate concentrations to bind AR and, along with NADPH, shift this enzyme to the activated state. GCK is also activated by micromolar concentrations of sorbitol, fructose, and fructose-1-phosphate in hepatocytes. The activation of AR may be cooperative between glucose, Glc6P, and NADPH, and the activated AR rapidly converts glucose to sorbitol. The two reaction polyol sequence is driven by the large potential energy difference of the combined [NADPH]/[NADP+] and [NAD+]/[NADH] coenzyme couples for the quantitative production of fructose. Fructokinase rapidly drives the production of fructose-1-phosphate that is funneled through the lower glycolysis to lactate with a Warburg effect. Elevated uric acid induces NOX4 to redirect citrate to the cytosol from the mitochondria to initiate DNL. The induction of HIF-1 alpha may stimulate reductive carboxylation to shift production of citrate and acetyl-CoA to the cytoplasm to support DNL. The alteration in ChREBP function activates the genes of DNL culminating in the increase and change in composition of liver fat, including triglycerides, diacylglycerol, ceramides, and fatty acids. Activation of this cascade depends on the gateway enzyme AR, where the coenzyme couples, large equilibrium constants, and thermodynamics ensure a wave of substrate for the synthesis of fat. Chronic activation of polyol-fructose-uric acid pathway may drive NAFLD and perhaps other metabolic diseases and depends as much on the glucose as it does the fructose.

## Vision statement

There is much to learn about the activation of AR in the liver. Careful enzymatic *in vitro* and *in vivo* work with liver homogenates and tissue culture are required to fully understand the details of AR activation. Of special interest are studies using the anomeric alpha, beta, and open forms of glucose including the search for an anomerase activity either as a functionality within AR or another enzyme that might be part of the activation process.

Additional studies are needed to determine the relationship between uric acid and NOX4 up-regulation, activation and translocation into the mitochondria. Moreover, once NOX 4 is in the mitochondria studies are needed to determine the way in which aconitase is inhibited.

Lastly, there is a need for studies to understand the relationship between LDH, IDH1, glutamine, and reductive carboxylation to provide cytoplasmic citrate versus the inhibition of the PDC and acetyl-CoA production in the mitochondria and how it relates to DNL when fructose metabolism is activated.

## Abbreviations

AMPD: AMP deaminase
AR: aldose reductase
ChREBP: carbohydrate response element-binding protein
DNL: de novo lipogenesis
FGF21: fibroblast growth factor 21
GCK: glucokinase
GKRP: glucokinase regulatory protein
Glc6P: glucose-6-phosphate
MCT1: monocarboxylate transporter I
NAFLD: non-alcoholic fatty liver disease
PPP: pentose phosphate pathway
VLDL: low-density lipoprotein
